# Physico-chemical, biochemical and nutritional characterisation of 42 organic wastes and residues from France

**DOI:** 10.1016/j.dib.2018.06.050

**Published:** 2018-06-22

**Authors:** Henry Fisgativa, Cyril Marcilhac, Romain Girault, Marie-Line Daumer, Anne Trémier, Patrick Dabert, Fabrice Béline

**Affiliations:** a Irstea, UR GERE, 17 av. de Cucillé, CS 64427, F-35044 Rennes, France; b UBL, Université Bretagne Loire, France

## Abstract

The data presented in this article regroup characterisation of organic matter and nutritional composition of 42 organic wastes and residues usually used as substrates for anaerobic digestion. Those wastes have different origins from agro-industrial, agricultural and urban sectors in France including: algae, slaughterhouse waste, fat, food waste, fruits and vegetables residues, green waste, slurry, manure, wastewater treatment plant sludge and agricultural residues. The properties of organic matter are distinguished between global parameters (pH, total solids, volatile solids, COD and BMP), organic matter fractionation (biochemical and Van Soest) and the main nutrients content (N, P, K, Mg, Ca and S).

**Specifications Table**

**Table:** 

Subject area	*Chemistry, Environmental Engineering*
More specific subject area	*Organic waste and residues characterisation*
Type of data	*Tables, tri-plot graphics, hierarchical clustering classifications*
How data was acquired	*Data was acquired using classical physico-chemical analyses and instruments including: pH probe, oven drying, furnace calcination, mineralisation, titration, ionic and gas chromatography, NMR*
Data format	*Raw data, statistical treatment*
Experimental factors	*After collection, each sample was stored at −20 °C until analyses. Frozen solid wastes were ground to obtain a homogenous sample. For nutrients analyses, a water extraction was performed on samples to obtain extracted phase and liquid samples were centrifuged in order to use the dissolved phase*
Experimental features	*Large waste and substrate characterisation with the aim to predict anaerobic digestion behaviour and digestate properties*
Data source location	*All organic wastes and residues samples were collected in France, mainly at Rennes (48°06′53″N 1°40′46″ W), Toulouse (43°36′16″N 1°26′38″E) and Montoldre (46°20′07″N 3°26′50″E)*
Data accessibility	*Data are available in this article*

**Value of the data**

• This data article provides a large characterisation of 42 organic waste and residues from France used for anaerobic digestion process.
• This data article focuses on characteristics useful to design anaerobic digestion and to predict biogas production and digestate characteristics according to substrate incorporation.
• The present data will be useful for comparison with other researches and for future studies in order to make correlations between physico-chemical and biochemical or nutritional characteristics.

## 1 Data

Table 1 listed the 42 organic waste and residues characterised in this article.

**Table 1:** Organic wastes and residues number, name and acronym.

**#**	**Substrate**	**Acronym**
**1**	**Seaweed**	SW1
**2**	**Freshwater Seaweed**	SW2
**3**	**Cattle Blood 1**	Blood1
**4**	**Cattle Blood 2**	Blood2
**5**	**Pig bristle**	PB
**6**	**Pig mucus**	PM
**7**	**Meat waste**	MW
**8**	**Sieving refusal 1**	SR1
**9**	**Sieving refusal 2**	SR2
**10**	**Slaughterhouse greases 1**	SG1
**11**	**Slaughterhouse greases 2**	SG2
**12**	**Slaughterhouse greases 3**	SG3
**13**	**Slaughterhouse greases 4**	SG4
**14**	**Municipal WWTP**^a^**grease 1**	MG1
**15**	**Municipal WWTP grease 2**	MG2
**16**	**Municipal WWTP primary sludge**	MPS
**17**	**Municipal WWTP secondary sludge 1**	MSS1
**18**	**Municipal WWTP secondary sludge 2**	MSS2
**19**	**Municipal WWTP secondary sludge 3**	MSS3
**20**	**Municipal WWTP secondary sludge 4**	MSS4
**21**	**Municipal WWTP secondary sludge 5**	MSS5
**22**	**Municipal WWTP secondary sludge 6**	MSS6
**23**	**Municipal WWTP secondary sludge 7**	MSS7
**24**	**Slaughterhouse WWTP secondary sludge**	SSS
**25**	**Dairy WWTP secondary sludge**	DSS
**26**	**Stercoral matter**	SM
**27**	**Cattle Manure 1**	M1
**28**	**Cattle Manure 2**	M2
**29**	**Cattle slurry**	CS
**30**	**Pig slurry 1**	Slurry1
**31**	**Pig slurry 2**	Slurry2
**32**	**Pig slurry 3**	Slurry3
**33**	**Wheat straw**	Straw
**34**	**Food waste 1**	FW1
**35**	**Food waste 2**	FW2
**36**	**Green waste 1**	GW1
**37**	**Green waste 2**	GW2
**38**	**Apple**	Apple
**39**	**Carrot 1**	Carrot1
**40**	**Carrot 2**	Carrot2
**41**	**Shallots pulps**	Shallot
**42**	**Onion pulps**	Onion

a WWTP: Wastewater treatment plant.

Table 2 shows global characteristics of 42 organic waste and residues including pH, total solids (TS), volatile solids (VS), chemical oxygen demand (COD) and the biochemical methane potential (BMP).

**Table 2:** Physicochemical characteristics of 42 waste and residues on wet weight basis.

**#**	**Substrate**	**pH**	**TS**	**VS**	**COD**	**BMP**
		**–**	**gTS.kgWW^−^**^**1**^	**gVS.kgWW**^**−1**^^a^	**gO**_**2**_**.kgWW**^**−1**^	**NLCH**_**4**_**.kgWW**^**−1**^
**1**	**SW1**	–	200.9	120.2	158.0	16.0
**2**	**SW2**	–	65.3	51.3	78.6	15.5
**3**	**Blood1**	7.4	155.3	139.9	211.8	67.3
**4**	**Blood2**	–	155.8	145.1	205.5	65.8
**5**	**PB**	–	285.5	278.4	404.7	77.2
**6**	**PM**	–	190.5	169.1	285.4	98.2
**7**	**MW**	–	330.3	293.9	646.7	256.0
**8**	**SR1**	–	176.3	161.6	277.0	79.6
**9**	**SR2**	–	328.6	310.8	499.2	169.8
**10**	**SG1**	7.3	237.7	231.6	754.9	200.5
**11**	**SG2**	–	557.1	552.6	1329.3	499.4
**12**	**SG3**	6.2	361.3	350.0	817.0	354.0
**13**	**SG4**	5.3	141.8	123.9	296.6	112.2
**14**	**MG1**	5.5	14.0	12.7	32.6	16.9
**15**	**MG2**	6.8	47.6	36.3	96.9	29.3
**16**	**MPS**	6.1	52.8	44.6	72.3	–
**17**	**MSS1**	6.5	57.0	45.8	75.2	11.7
**18**	**MSS2**	5.6	42.0	34.4	45.0	11.7
**19**	**MSS3**	6.9	60.3	47.8	75.4	12.4
**20**	**MSS4**	6.5	61.6	41.3	79.8	8.5
**21**	**MSS5**	7.0	69.2	40.0	75.5	11.3
**22**	**MSS6**	6.8	45.3	31.2	47.6	–
**23**	**MSS7**	6.8	5.9	4.2	8.5	1.2
**24**	**SSS**	7.4	20.9	12.9	18.9	4.7
**25**	**DSS**	7.4	9.3	4.5	6.6	1.7
**26**	**SM**	–	166.3	155.4	231.1	52.5
**27**	**M1**	9.0	276.5	241.2	303.0	89.4
**28**	**M2**	–	225.2	204.5	279.1	57.0
**29**	**CS**	8.6	106.9	79.2	121.8	18.9
**30**	**Slurry1**	7.8	56.8	39.7	57.3	9.3
**31**	**Slurry2**	7.6	38.6	25.0	39.9	3.7
**32**	**Slurry3**	7.6	73.4	53.3	95.2	25.1
**33**	**Straw**	7.5	904.7	870.7	1177.0	294.6
**34**	**FW1**	5.8	259.1	219.9	412.0	111.9
**35**	**FW2**	–	271.3	257.5	416.3	147.2
**36**	**GW1**	6.3	261.4	215.5	344.0	71.5
**37**	**GW2**	–	235.1	192.9	307.4	54.3
**38**	**Apple**	3.9	109.9	107.1	138.0	48.3
**39**	**Carrot1**	5.4	97.1	89.9	125.8	39.3
**40**	**Carrot2**	–	177.7	167.2	196.8	55.3
**41**	**Shallot**	–	226.7	212.7	254.6	87.9
**42**	**Onion**	–	211.5	194.6	228.5	81.1

a WW – Wet weight.

Two kinds of organic matter fractionation data are shown in Table 3. On the one hand, the biochemical fractionation discriminates organic matter into lipids, proteins and carbohydrates and on the other hand the Van Soest fractionation describes water and neutral detergent soluble organic matter (SOL), hemicellulose-like fractions (HEM), cellulose-like fractions (CEL), lignin-like fractions (LIG).

**Table 3:** Biochemical and Van Soest fractionation of organic matter of 42 organic waste and residues.

**#**	**Substrate**	**Biochemical fractionation**			**Van Soest fractionation**			
		**Lipids**	**Proteins**	**Carbohydrates**	SOL^*^	HEM^*^	CEL^*^	LIG^*^
		**%VS**	**%VS**	**%VS**	**%VS**	**%VS**	**%VS**	**%VS**
**1**	**SW1**	2.9	22.5	74.5	–	–	–	–
**2**	**SW2**	2.8	47.7	49.6	–	–	–	–
**3**	**Blood1**	7.5	92.5	0.0	98.7	1.0	0.2	0.1
**4**	**Blood2**	1.9	98.1	0.0	9.7	79.1	7.6	3.6
**5**	**PB**	1.5	93.3	5.1	24.1	14.5	0.4	61.0
**6**	**PM**	23.7	76.3	0.0	–	–	–	–
**7**	**MW**	58.2	41.8	0.0	83.8	13.7	0.6	2.0
**8**	**SR1**	15.4	24.4	60.2	24.7	25.3	30.0	20.0
**9**	**SR2**	25.4	37.2	37.4	39.2	24.3	15.6	20.8
**10**	**SG1**	78.4	4.8	16.8	89.4	2.5	6.5	1.6
**11**	**SG2**	85.1	9.7	5.2	94.7	2.8	1.3	1.2
**12**	**SG3**	91.3	7.1	1.6	92.4	5.7	-0.5	2.4
**13**	**SG4**	60.3	12.4	27.3	68.4	15.6	9.6	6.3
**14**	**MG1**	59.2	8.4	32.5	87.9	5.5	3.6	3.0
**15**	**MG2**	22.6	23.5	53.9	–	–	–	–
**16**	**MPS**	12.9	0.0	87.1	–	–	–	–
**17**	**MSS1**	10.5	58.6	30.9	60.3	27.8	3.1	8.8
**18**	**MSS2**	–	–	–	–	–	–	–
**19**	**MSS3**	–	–	–	–	–	–	–
**20**	**MSS4**	5.1	42.7	52.2	66.8	14.7	6.5	12.0
**21**	**MSS5**	7.6	67.4	25.0	–	–	–	–
**22**	**MSS6**	–	–	–	–	–	–	–
**23**	**MSS7**	4.7	76.2	19.2	79.9	18.1	0.3	1.7
**24**	**SSS**	–	–	–	–	–	–	–
**25**	**DSS**	11.5	68.9	19.5	58.4	23.1	5.4	13.1
**26**	**SM**	12.5	64.8	22.7	36.3	28.1	18.4	17.2
**27**	**M1**	10.8	73.0	16.2	70.3	25.1	2.6	2.1
**28**	**M2**	9.1	13.5	77.4	21.6	28.6	35.4	14.3
**29**	**CS**	8.6	9.9	81.5	23.1	28.3	25.8	22.8
**30**	**Slurry1**	4.8	7.5	87.6	–	–	–	–
**31**	**Slurry2**	6.6	19.1	74.3	40.6	21.8	21.9	15.6
**32**	**Slurry3**	13.9	27.6	58.5	27.1	30.8	23.1	19.0
**33**	**Straw**	4.5	32.6	62.9	41.0	19.3	17.3	22.3
**34**	**FW1**	8.2	0.0	91.8	–	–	–	–
**35**	**FW2**	7.7	2.3	90.0	10.2	34.5	35.2	20.1
**36**	**GW1**	26.4	22.6	50.9	51.5	35.9	10.8	1.8
**37**	**GW2**	22.2	23.7	54.2	–	–	–	–
**38**	**Apple**	11.9	22.2	65.9	35.5	34.2	22.5	7.8
**39**	**Carrot1**	6.6	26.5	66.9	–	–	–	–
**40**	**Carrot2**	3.3	3.9	92.8	89.6	3.6	1.1	5.7
**41**	**Shallot**	15.2	8.2	76.6	88.2	3.1	6.6	2.2
**42**	**Onion**	2.7	9.7	87.6	44.3	16.9	33.0	5.7

SOL – water and neutral detergent soluble, HEM – hemicellulose-like, CEL – Cellulose-like, LIG – Lignin-like.

**Fig. 1:**
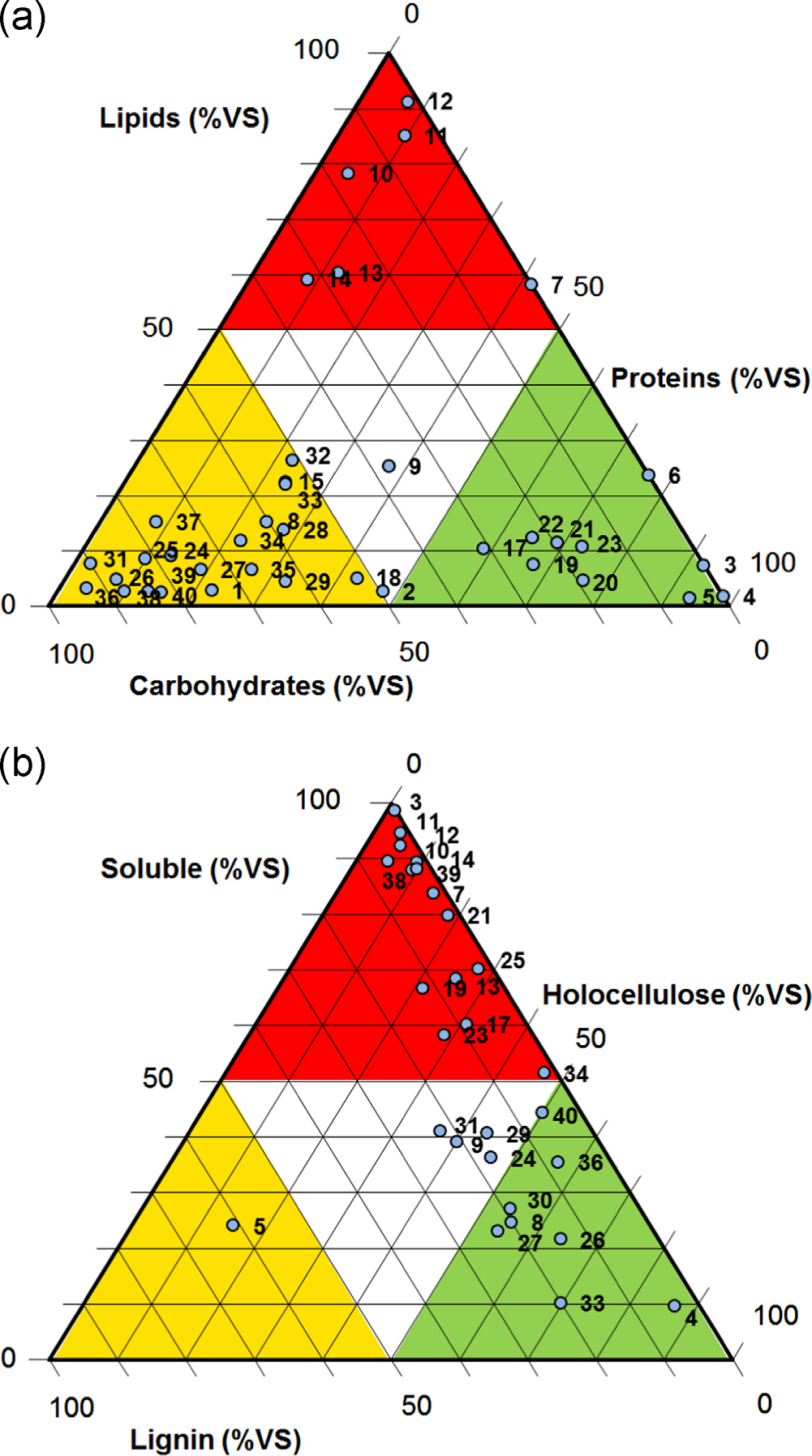
Tri-plots graphics of (a) biochemical and (b) Van Soest fractionation of organic matter of 42 waste and residues. Number of wastes and substrates are indicated in Table 1. Holocellulose is the sum of hemicellulose-like and cellulose-like fractions.

Fig. 1 shows tri-plots graphics of both methods results which help to identify the global fractionation data of each studied waste.

Table 4 shows data of nitrogen, phosphorus, potassium, magnesium, calcium and sulphur of 42 organic waste and residues expressed on wet weight basis (WW). These measurements were performed on the extracted or dissolved phases depending of total solids content of waste.

**Table 4:** Main nutrients (N, P, K, Mg, Ca and S) content of 42 waste and residues on wet weight basis.

**#**	**Substrate**	**TKN**	**NH**_**4**_^**+**^	**P**	**K**	**Mg**	**Ca**	**S**
		**g.kgWW**^−^^**1**^	**g.kgWW**^−**1**^	**g.kgWW**^**−1**^	**g.kgWW**^**−1**^	**g.kgWW**^**−1**^	**g.kgWW**^−^^**1**^	**g.kgWW**^–^^**1**^
**1**	**SW1**	4.7	0.3	–	–	–	–	–
**2**	**SW2**	3.9	0.1	–	–	–	–	–
**3**	**Blood1**	22.2	0.2	0.2	0.5	0.3	0.4	0.9
**4**	**Blood2**	23.7	0.6	0.2	0.4	0.0	0.1	1.1
**5**	**PB**	41.3	–	1.1	0.4	0.3	1.5	7.4
**6**	**PM**	21.2	–	1.3	6.4	0.2	0.1	1.5
**7**	**MW**	17.6	–	1.3	1.1	1.1	0.9	1.6
**8**	**SR1**	6.4	–	0.9	0.3	0.2	2.0	0.5
**9**	**SR2**	17.8	–	0.8	0.5	0.4	2.6	1.7
**10**	**SG1**	1.7	0.1	0.3	0.1	0.1	1.9	0.2
**11**	**SG2**	9.1	–	0.6	0.3	0.0	0.4	0.8
**12**	**SG3**	4.0	0.1	0.3	0.2	0.0	0.5	0.5
**13**	**SG4**	2.7	0.2	0.4	0.2	0.1	2.3	0.2
**14**	**MG1**	0.2	0.0	0.1	0.1	0.1	0.3	–
**15**	**MG2**	1.3	0.1	0.3	0.1	0.1	1.9	–
**16**	**MPS**	0.0	0.2	0.5	0.1	0.1	1.0	–
**17**	**MSS1**	4.6	0.1	1.9	0.7	0.4	0.9	–
**18**	**MSS2**	0.1	–	–	–	–	–	–
**19**	**MSS3**	3.6	0.4	1.8	0.5	0.4	1.4	0.5
**20**	**MSS4**	4.6	0.1	3.0	0.9	0.3	0.3	0.3
**21**	**MSS5**	5.1	0.1	5.2	2.0	0.3	5.3	0.5
**22**	**MSS6**	–	0.1	1.8	0.4	0.3	0.9	–
**23**	**MSS7**	0.5	0.0	0.1	0.1	0.1	0.1	–
**24**	**SSS**	1.5	0.1	0.3	0.2	0.1	0.2	0.1
**25**	**DSS**	0.6	0.0	0.5	0.1	0.1	0.5	–
**26**	**SM**	3.3	–	0.8	0.8	0.1	0.8	0.2
**27**	**M1**	4.6	0.8	1.0	6.6	0.8	2.4	0.6
**28**	**M2**	2.6	–	1.4	1.6	0.4	1.2	0.3
**29**	**CS**	4.5	2.1	0.7	5.1	0.6	2.1	–
**30**	**Slurry1**	4.4	2.6	1.2	2.8	0.8	2.0	0.2
**31**	**Slurry2**	4.3	3.0	0.9	2.4	0.5	1.9	–
**32**	**Slurry3**	3.2	3.9	1.4	5.1	0.8	1.9	–
**33**	**Straw**	3.2	0.0	0.5	6.8	0.5	1.9	0.6
**34**	**FW1**	8.0	0.1	2.0	2.7	0.2	9.2	0.8
**35**	**FW2**	10.7	0.1	1.0	2.4	0.2	0.5	–
**36**	**GW1**	8.1	0.4	0.8	5.0	0.4	2.1	0.6
**37**	**GW2**	8.5	0.1	1.9	11.6	1.0	2.8	–
**38**	**Apple**	0.7	0.0	0.1	1.1	0.3	0.1	–
**39**	**Carrot1**	1.2	0.0	0.3	3.2	0.1	0.4	0.2
**40**	**Carrot2**	2.6	–	0.5	2.9	0.3	1.1	0.2
**41**	**Shallot**	5.2	–	0.5	3.2	0.3	2.6	0.8
**42**	**Onion**	4.1	–	0.4	2.4	0.3	3.9	0.6

**Fig. 2:**
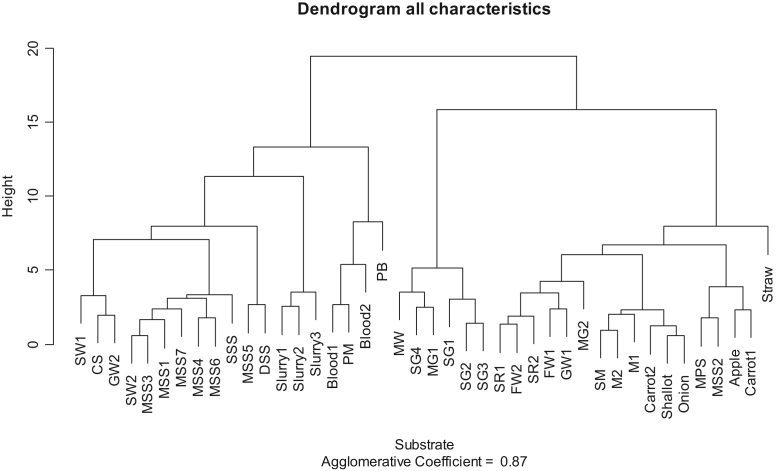
Hierarchical clustering from all characteristics expressed on total solids basis.

**Fig. 3:**
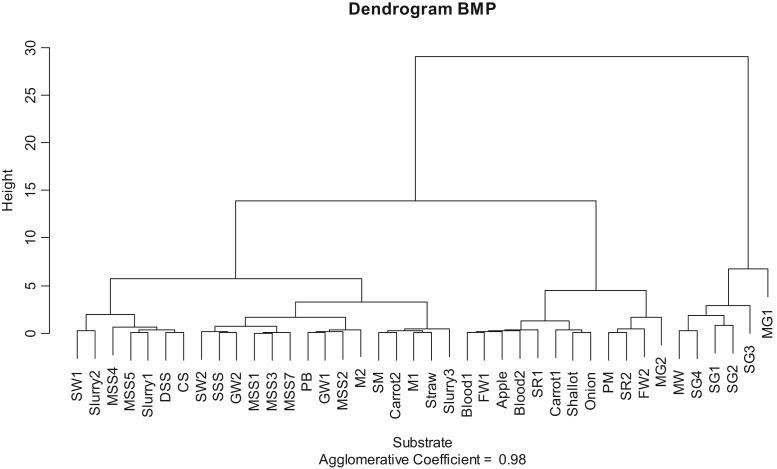
Hierarchical clustering from BMP expressed on total solids basis.

**Fig. 4:**
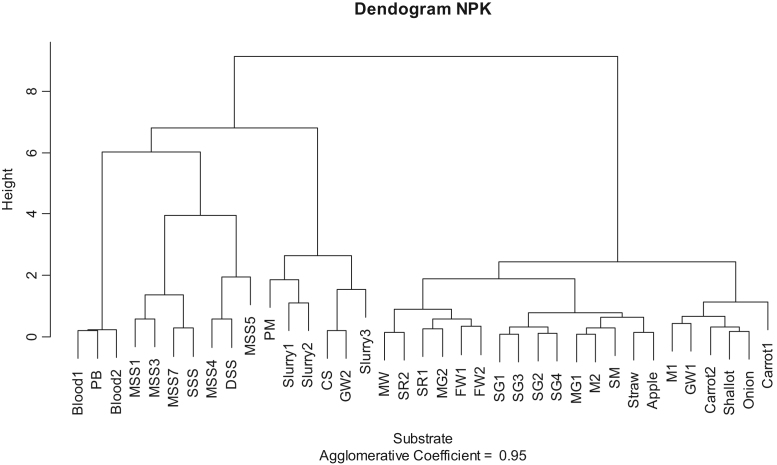
Hierarchical clustering from NPK expressed on total solids basis.

Fig. 2, Fig. 3, Fig. 4 show hierarchical clustering classifications performed using software R 3.4.3 (cluster and FactoMineR packages). All characteristics values units were expressed on total solids basis (g.kgTS^−1^ or NLCH_4_.kgTS^−1^), excepted for TS (g.kgWW^−^^1^) and pH (pH units).

Dendogram obtained from all characteristics (Fig. 2) shows two main classes depending on total solids content (liquid versus solid substrate). When the statistical analysis is performed only on BMP values (Fig. 3), substrates with high BMP content (mainly high fat content substrates) are classified separately of low BMP content substrates. Latter, among low BMP content substrates, a classification between very low BMP (average of 227 NLCH_4_.kgTS^−^^1^) corresponding mainly to substrates already degraded (slurry, sludge, …) and average BMP (average of 462 NLCH_4_.kgTS^−^^1^) corresponding to raw substrates. Finally, the analysis performed on NPK characteristics (Fig. 4) shows initial first stage of classification between substrates with high nutrient contents (mainly animal residues, sewage sludge and slurry) and those with low nutrient contents (mainly high carbohydrate content substrates). Latter, among high nutrient content substrates, a classification between high nitrogen content substrates (as blood), high K content substrates (as slurry) and high P content substrates (as sewage sludge) may be assessed.

## 2 Experimental design, materials and methods

### 2.1 Collection and preparation of samples

The 42 wastes and residues were collected from different agro-industrial, agricultural or urban sources. The solid substrates followed a method of quartering in order to obtain a representative sample. The sampled solids were separately ground into liquid nitrogen to a size of around 1–3 mm to obtain a homogenous sample. Floating solids from municipal WWTP grease were separated, and then it was mixed with the liquid phase before sampling to increase the homogeneity. Each substrate was stored at –20 °C prior to analysis.

### 2.2 Physicochemical and biochemical analysis

pH was measured directly on liquid samples. For solid samples, 60 g of sample were diluted in 300 ml of water, agitated during 1 h and pH was measured after 3 h of decantation. TS, VS and COD were determined following standard methods (EN12880-12879, NF T90-101).

Biochemical methane potential (BMP) was determined as described by Fisgativa et al. [1]. Measurements were made in triplicate using hermetically closed 572 ml-bottles, mixing 40 ml of inoculum and 1–95 g of liquid substrate or ground substrate in the case of solid sample, to raise a 1 gVS inoculum:1 gVS substrate ratio. The inoculum was obtained from a well-established anaerobic digester (100 L) acclimated to degrade pig slurry supplemented with horse feed. The bottles were incubated at 38 °C for about 40 days. Daily pressure measurements enabled quantification of biogas production. Biogas was then sampled to determine CH_4_ concentration using a gas chromatography equipped with an electron capture detector (Agilent Technologies, 6890N, USA) according to the method as described by Lucas et al. [2].

Organic matter fractionation was performed in two ways: the total lipids, proteins and carbohydrates fractionation (biochemical fractionation) and the Van Soest fractionation. To determine lipids content, a nuclear magnetic resonance (NMR) measurement was performed as described by Picard et al. [3]. The analysis was carried out on the solid fraction after it was dried at 38 °C until constant weight and grounded at 1.5 mm. In brief, lipids measurements were performed with a low field NMR operating at a frequency of 10 MHz using a Brucker spectrometer Minispec MQ 10. About 1.5 g of dried sample was placed in a 30 mm-diameter NMR tube with an approximate height of 10 mm. The calibration equation of the NMR apparatus was calculated with four reference tubes filled with different heights of colza oil (CAS 8002139) between 1 and 10 mm. For each sample and for each reference tube, the free induction decay was measured for about 45 s using a relaxation delay of 3 s and 9 scans accumulation. Lipid content was then calculated on the basis of a simple linear regression [4]. Protein content was calculated using the nitrogen content as described by Dintzis et al. [5]:

$$\text{Proteins}=\left(\text{TKN}-{{\mathrm{NH}}_{4}}^{+}\right)*6.25$$

where Proteins, TKN and NH_4_^+^ are expressed in g N.kgWW^−^^1^ and 6.25 is the average conversion factor to estimate the protein content based on a N analysis. Then, carbohydrates content was considered being the residual fraction of VS outwards the sum of lipids and proteins.

The dried fraction used to determine lipids content was also used for the modified Van Soest fibres analysis method, as described by Fisgativa et al. [1]. In this method, successive extractions with neutral detergent (NDF), acid detergent (ADF) and lignin acid detergent (ADL) are used to discretise the non-soluble organic matter into 5 fractions: water soluble OM (SOL_W_) (determined as the difference between water extracted VS and raw waste VS), soluble in neutral detergent (SOL_NDF_), hemicellulose-like OM (HEM), cellulose-like OM (CEL) and lignin-like OM (LIG). In this study the SOL_W_ and SOL_NDF_ were summarised in a unique SOL fraction (water and neutral detergent OM fraction).

Total NTK, total NH_4_^+^, total phosphorus and total potassium were determined with the standard methods (NF EN 13342, NF EN ISO 11885). Cations and anions were analysed using a Metrohm 850 Professional Ionic Chromatography on liquid samples and dissolved phase.

## References

[1] Fisgativa H., Tremier A., Le Roux S., Bureau C., Dabert P. (2017). Understanding the anaerobic biodegradability of food waste: relationship between the typological, biochemical and microbial characteristics. J. Environ. Manag..

[2] Lucas T., Le Ray D., Peu P., Wagner M., Picard S. (2007). A new method for continuous assessment of CO_2_ released from dough baked in ventilated ovens. J. Food Eng..

[3] S. Picard, F. Beline, R. Girault, G. Bridoux, C. Cambert, A. Davenel, Determination of lipid fraction from organic wastes using Nuclear Magnetic Resonance (NMR): comparison to the soxhlet method, in: Proceedings of the 13th World Congress on Anaerobic Digestion, Santiago de Compostela, Spain, 2014.

[4] Toussaint C.A., Medale F., Davenel A., Fauconneau B., Haffray P., Akoka S. (2002). Determination of the lipid content in fish muscle by a self-calibrated NMR relaxometry method: comparison with classical chemical extraction methods. J. Sci. Food Agric..

[5] Dintzis F.R., Cavins J.F., Graf E., Stahly T. (1988). Nitrogen-to-protein conversion factors in animal feed and fecal samples. J. Anim. Sci..

