# Supplementary material for: Physico-chemical, biochemical and nutritional characterisation of 42 organic wastes and residues from France
Source: Data Brief. 2018 Jun 22;19:1953–62. doi: 10.1016/j.dib.2018.06.050 (PMC6141762; doi:10.1016/j.dib.2018.06.050)
Supplement: Supplementary file 1 — Supplementary material [file mmc1.docx]

**Conflicts of interest:**

The seven authors confirm that there are no known conflicts of interest associated with this publication and there has been no significant financial support for this work that could have influenced its outcome.
